# Molecular detection and assessment of the epidemiological risk factors associated with equine herpesvirus 2 and 5 in working equids in central Ethiopia

**DOI:** 10.1002/vms3.925

**Published:** 2022-09-05

**Authors:** Kifle Wondimagegnehu, Samson Leta, Kebede Amenu, Haileleul Negussie

**Affiliations:** ^1^ Alage Agricultural Technical Vocational Education and Training College Ministry of Agriculture Alage Ethiopia; ^2^ College of Veterinary Medicine and Agriculture Addis Ababa University Bishoftu Ethiopia

**Keywords:** EHV‐2, EHV‐5, Ethiopia, equids, PCR, respiratory disease

## Abstract

**Background:**

Respiratory disease is the most common presenting complaint at veterinary clinics and a priority concern for equid owners and veterinary practitioners in Ethiopia.

**Objectives:**

This study aimed to report the molecular detection of EHV‐2 and EHV‐5 and to assess the risk factors associated with infection in working equids in central Ethiopia.

**Methods:**

Nasopharyngeal swabs were collected from 58 horses and donkeys to detect EHV‐2 and EHV‐5 using PCR targeting the conserved region of glycoprotein B (gB) genes.

**Results:**

From 58 equids, EHV‐5 and EHV‐2 were detected in 20 (34.5%) and 19 (32.8%) equids, respectively. Concurrent infection with EHV‐2 and EHV‐5 was found in 6 (10.3%) equids who exhibited respiratory clinical signs. EHV‐2 was detected in a significantly higher (*p* = 0.002) proportion of horses (54.5%; *n* = 18) than donkeys (4%; *n* = 1). In contrast, EHV‐5 was detected in a significantly higher (*p* = 0.004) proportion of donkeys (56%; *n* = 14) compared to horses (18.2% *n* = 6). EHV‐2‐positive equids were seven times more likely to display clinical signs of respiratory disease than EHV‐2‐negative equids (Odds ratio (OR) = 6.9; 95%CI: 1.72‐27.60). However, statistically significant (*p* = 0.832) difference was not observed for EHV‐5. EHV‐2 was detected in a significantly higher (*p* = 0.004) proportion of female (50%; *n* = 16) compared to male equids (11.5%; *n* = 3).

**Conclusions:**

This study revealed the molecular detection of EHV‐2 and EHV‐5 in horses and donkeys residing in central Ethiopia. The association between EHV‐2‐test‐positive equids and displaying of clinical signs of respiratory disease was observed, which suggests EHV‐2 involvement in the development of respiratory disease; however, it deserves further investigation.

## Graphical abstract

This study revealed the molecular detection of EHV‐2 and EHV‐5 in horses and donkeys in central Ethiopia. We demonstrated that the association between EHV‐2‐test‐positive equids and displaying of clinical signs of respiratory disease, which suggests EHV‐2 involvement in the development of respiratory disease.

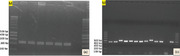

## 1 INTRODUCTION

Approximately 60% of the world's horse population and over 95% of all donkeys and mules are found in developing countries (Pritchard et al., 2005). Equids have an essential role in the livelihoods of millions of people in Ethiopia mainly used for transportation, agricultural purposes and other social values. Notwithstanding basic husbandry and welfare needs, infectious diseases compromise the health and welfare of working equids in Ethiopia. Among the multiple infectious disease problems affecting working equids, equine herpesviruses are one of the major viral respiratory pathogens that threaten the health of equids (Getachew et al., 2014).

Equine herpesviruses (EHVs) are enveloped DNA viruses that have a major economic and welfare impact on the horse industry. Equine herpesvirus 2 (EHV‐2) and equine herpesvirus 5 (EHV‐5) are classified within the genus *Percavirus* of the family of *Gammaherpesvirinae*, which are recognised as infecting horses as their natural host (Davison et al., 2009; King et al., 2012). EHV‐2 and EHV‐5 contain a range of immunomodulatory genes that interact with the immune system of the host, and this is suggested to compromise host immunity and increase the susceptibility of the host to opportunistic infections (Dunowska et al., 2002; Gilkerson et al., 2015; Nordengrahn et al., 1996). Epidemiologic studies of these viruses are complicated by the high rate of infection in the population and the range of clinical signs associated with the detection of these viruses (Gilkerson et al., 2015). Infections caused by EHV‐2 and EHV‐5 usually occur during the early stages of the life of horses. These viruses can establish lifelong latency in a large proportion of equids that ensures their survival in equine populations and enables the virus to be shed sporadically throughout the lifetime of the host (Hartley et al., 2013). Horses with latent and/or active infection harbour these viruses and serve as carriers and reservoirs of infections. These carriers become disseminators of the viruses when they are stressed (transported, raced, starved, etc.) (Muscat et al., 2018) and immune‐compromised (Ma et al., 2013).

Clinical disease associated with EHV‐2 and ‐5 has been difficult to establish (Hartley et al., 2013), as both viruses have been detected in samples from clinically healthy equids as well as those exhibiting clinical signs of respiratory disease (Bell et al., 2006; Dunowska et al., 2002; Wang et al., 2007). However, previous reports suggested that they are associated with certain disease complaints. EHV‐2 has been implicated with upper respiratory disease, keratoconjunctivitis, pneumonia, chronic follicular pharyngitis and fever (Allen and Murray, 2004; Fortier et al., 2010). EHV‐5 typically causes upper respiratory tract disease (e.g. pharyngitis) or keratoconjunctivitis accompanied by clinical signs such as nasal and ocular discharge, tachypnea, coughing, fever, enlarged lymph nodes, anorexia, poor body condition and depression (Dunowska et al., 2002; Rushton et al., 2013). EHV‐5 has also been implicated as an etiologic agent for fatal equine multinodular pulmonary fibrosis (EMPF) (Dunowska et al., 2014; Williams et al., 2007). The high correlation between the presence of EMPF and EHV‐5 DNA suggested that the virus is involved in the development of lung fibrosis (Van Cleemput et al., 2019). However, so far, the exact pathogenic role played by EHV‐2 and EHV‐5 is uncertain.

Respiratory pathogens are important causes of disease in equine populations worldwide. Previous studies conducted in Ethiopia have documented that respiratory disease particularly coughing and nasal discharge is one of the major health concerns for working equids in Ethiopia (Laing et al., 2018). Equine herpesviruses are important pathogens that are involved in respiratory diseases of varying severity (Negussie et al., 2015; Negussie et al., 2017). Epidemiological studies have reported that the severity of the diseases associated with EHV‐2 and EHV‐5 is often influenced by various risk factors such as the age, physical condition, immune status and breed of the host (Agnol et al., 2016; Stasiak et al., 2018). However, little is known about the disease burden and the epidemiological risk factors associated with the disease in Ethiopian working equids. Therefore, it is important to have knowledge on the epidemiology of equine gammaherpesviruses for a better understanding of the impact of the disease on the well‐being of the working equids. The study was conducted in areas where respiratory diseases in working equids were a priority concern for equine owners and veterinary practitioners. Respiratory disease was the most common presenting complaint at veterinary clinics in the midland and highland areas of central Ethiopia (Getachew et al., 2014; Laing et al., 2018, 2016). Despite this, there is little study on equine gammaherpesviruses in the working equid population. Thus, this study aimed to report the molecular detection of EHV‐2 and EHV‐5 and to assess the risk factors associated with infection in working equids in central Ethiopia.

## 2 METHODS

### 2.1 Study areas

**FIGURE 1:**
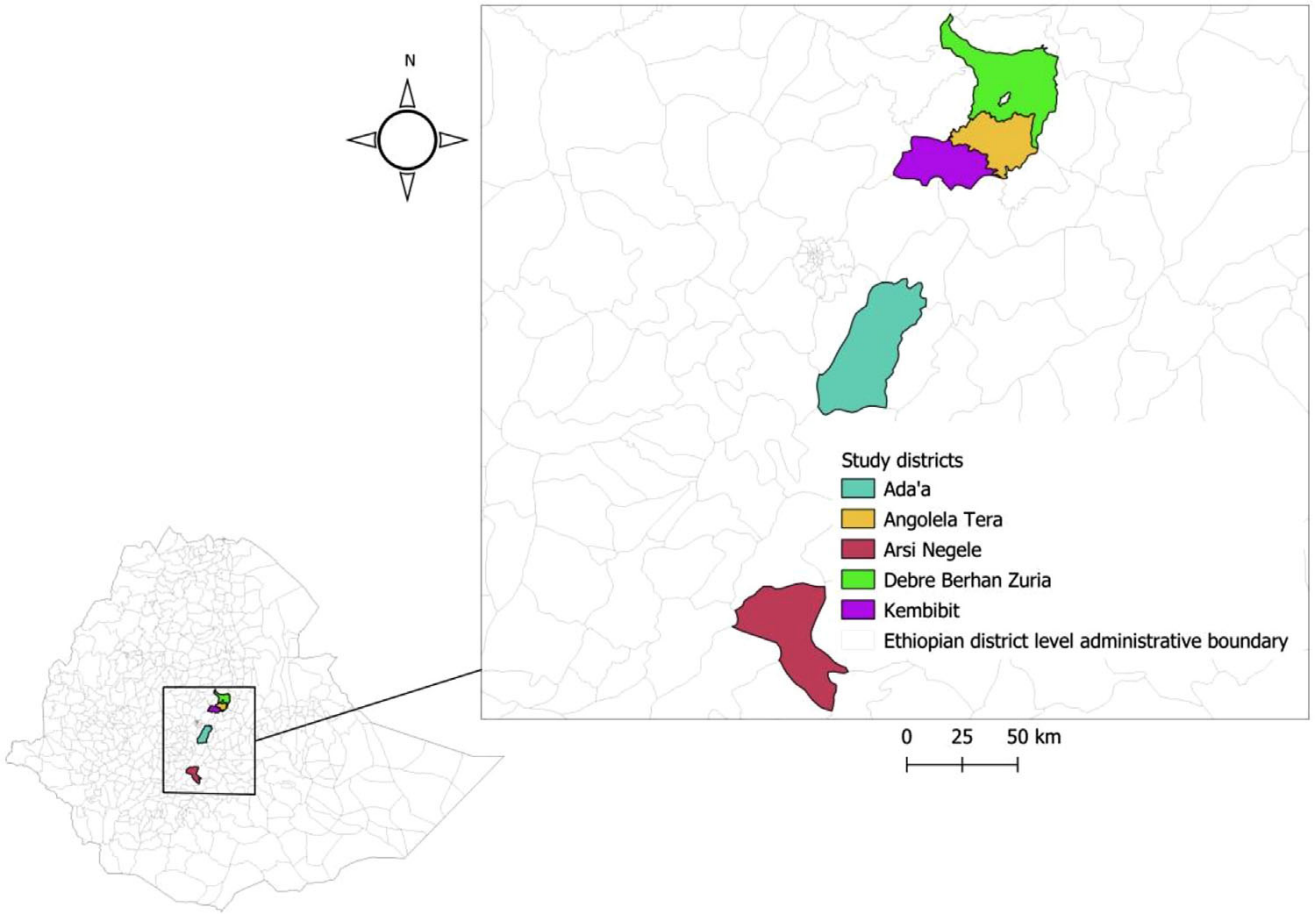
Map of Ethiopia showing the study sites: Add'a, Angolelana Tera, Arsi Negele, Debre Berhan zuria (Basona Werana) and Kimbibit. This map was developed from Ethiopian's Districts Administrative boundaries shapefile 2021 using QGIS version 3.1.1.2

The study was conducted in areas where respiratory disease is the most common presenting complaint at veterinary clinics in central Ethiopia. This study was conducted in Angolelana Tera (Chacha), Basona Werana (Debre Berhan Zuria), Kembebit (Sheno), Bishoftu (Ade'a) and Arsi Negele districts (Figure 1). These districts were selected based on previous respiratory disease reports and the presence of high equine population (CSA, 2020; Laing et al., 2018, 2016).

Samples were collected from five districts of different altitudes. The locations of the sampling sites were recorded by a global positioning system (GPS). The study areas consist of two agroecological zones: highlands (above 2500 m above sea level (masl) and midland (between 1500 and 2500 masl). The study was conducted in the highlands of Angolelana Tera district with an altitude of 2812 masl and in the midlands of Basona Werena (Debre Berhan zuria) district with an altitude of 2360 masl in the Amhara Regional State. The study was also conducted in Kembibit, Ada'a (Bishoftu), and Arsi Negele districts of Oromia Regional State of Ethiopia, which are situated with an altitude of 2630–3020 masl, 1900 masl and 1500–2300 masl, respectively.

### 2.2 Sample collection

In each study site, equids were clinically examined for evidence of respiratory distress, coughing and nasal discharge. Equids without noticeable respiratory clinical signs were also included in this study. During sampling, information such as age, sex, species of the host, presence of clinical signs and geographical locations were also collected to determine whether viral detection was associated with potential risk factors. A total of 58 equids composed of 25 donkeys and 33 horses were enrolled in this study. The study population was also categorised into three age groups: ≤3 years (yearlings and young equids), 4–10 years (adults) and above 10 years (old).

Nasopharyngeal swabs were collected from equids with clinical signs of respiratory disorder and from those equids without noticeable clinical signs. Nasopharyngeal swabs were collected using standard Sigma Virocult® swab – 15 cm long (Corsham, Wiltshire, UK) with a cellular foam bud. Each collected swab was placed into 3 ml viral transport medium (VTM) containing an equal amount of glycerol and 0.04 M of phosphate‐buffered saline (PBS) supplemented with 1 μg/ml gentamycin (Invitrogen, Paisley, UK), 1 mg/ml streptomycin (Certa, Braine l'Alleud, Belgium), 1 mg/ml kanamycin (Sigma, St. Louis, MO, USA), 1000 U/ml penicillin (Continental Pharma, Puurs, Belgium) and 5 μg/ml amphotericin B (Bristol‐Myers Squibb, New York, USA). Epidemiological information and disease conditions were also recorded during sampling. All collected samples were labelled and immediately placed in a cooler containing ice and transported to the National Veterinary Institute, Bishoftu. Samples were kept at –20°C until further processing.

### 2.3 DNA extraction and polymerase chain Reaction (PCR) assay

Total DNA extraction was performed from 200 μl of nasopharyngeal swabs using the QIAamp DNA Mini Kit (Qiagen, Hilden, Germany) according to the manufacturer's instructions. The extracted DNA was stored at –20°C until processed. PCR amplification was performed using virus‐specific primers targeting the conserved region of glycoprotein B (gB) genes for EHV‐2 (444 bp) and EHV‐5 (293 bp) according to (Diallo et al., 2008) and (Holloway et al., 1999), respectively (Table 1).

**TABLE 1:** Primers used for amplification of a region of the gB genes of EHV‐2 and EHV‐5

Virus	Region	PCR primers	Size	Reference
EHV‐2	gB	FW: 5′GCCAGTGTCTGCCAAGTTGATA‐3′ RV: 5′‐CATGGTCTCGATGTCAAACACG‐3′	444 bp	Diallo et al.(2008)
EHV‐5	gB	FW: 5′‐ATGAACCTGACAGATGTGCC‐3′ RV: 5′‐CACGTTCACTATCACGTCGC‐3′	293 bp	Holloway et al. (1999)

Each PCR was performed using Agilent's Herculase II fusion DNA polymerase (Agilent Technologies, Inc., Santa Clara, CA, USA). Each reaction was processed in a total volume of 25 μl mixture containing 12.5 μl of nuclease‐free water, 5 μl of Herculase reaction buffer, 0.5 μl Herculase II fusion DNA polymerase, 0.5 μl of 25 mM of each deoxynucleoside triphosphate (dNTP) mix, 1 μl of each forward and reverse primers, 2.5 μl of dimethyl sulphoxide (DMSO), and 2 μl template DNA with a concentration of 10 ng/μl. In each reaction, nuclease‐free water was added as a negative control, and known EHV‐2 and EHV‐5 DNA extract (10 ng/μl) was used as a positive control. The positive controls were used from our virus stock that has been isolated and sequenced in 2017.

PCR assay targeting the region of gB genes of EHV‐2 and EHV‐5 was amplified using the following thermocycling conditions: an initial denaturation step of 95°C for 5 min, followed by 40 cycles of amplification, using denaturation at 95°C for 30 s, annealing at 60°C and extension at 72°C for 45 s and followed by a final extension at 72°C for 10 min. The final PCR products were visualised on a 1.5% agarose gel electrophoresis using electrolyte buffer Tris Acetate EDTA (TAE) and stained with gel red. Then, 1 μl of 6× loading buffer was added to 5 μl PCR products and loaded into the wells. The amplified DNA was examined using a UV transilluminator for specific size bands (444 bp for EHV‐2 and 293 bp for EHV‐5).

### 2.4 Statistical analysis

Data generated from laboratory investigations were recorded using Microsoft Excel spreadsheets and analysed using STATA version 14 for Windows (Stata Corp. College Station, TX, USA). Logistic regression analysis was employed to investigate associations between risk factors and EHV‐2/5 detection. Differences were considered statistically significant when *p* value was < 0.05.

## 3 RESULTS

**FIGURE 2:**
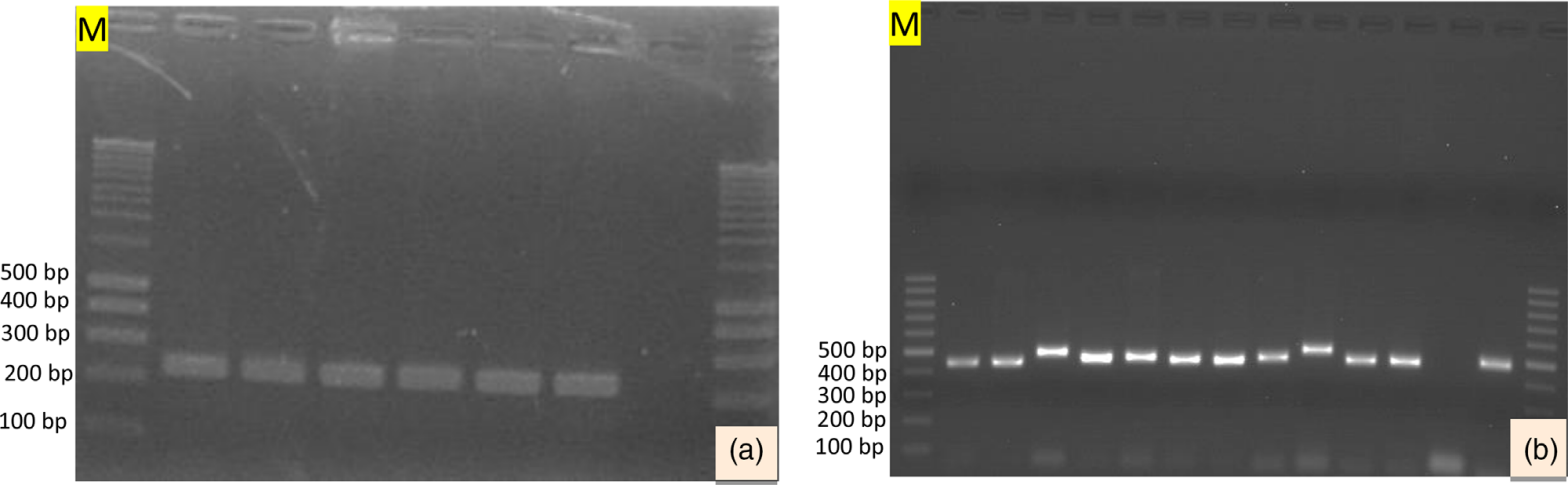
Representative agarose gel electrophoresis of DNA amplification product generated by targeting specific regions of EHV‐5 gpB gene (293 bp) (a) and EHV‐2 gpB gene (444 bp) (b) on 1.5% agarose gel with a DNA Molecular Weight Marker (M) of 100 bp

Virus‐specific PCR were used to detect EHV‐2 and EHV‐5 from 58 nasopharyngeal swabs collected from horses and donkeys. From a total of 58 equids, 19 (32.8%) and 20 (34.5%) equids were found positive for EHV‐2 (Table 2 and Figure 2) and EHV‐5 (Table 3 and Figure 2), respectively. EHV‐2 was detected in a significantly higher proportion (*p* = 0.002) of horses (54.5%; *n* = 18) than donkeys (4%; *n* = 1) (Table 2). In contrast, EHV‐5 was detected in a significantly higher (*p* = 0.004) proportion of donkeys (56%; *n* = 14) compared with horses (18.2%; *n* = 6) (Table 3).

**TABLE 2:** Multivariable logistic regression analysis of risk factors associated with EHV‐2 detection

Variables	No. of equids	EHV‐2 positive (%)	OR	95% Confidence interval	*p* Value
Species					
Donkey	25	1 (4.0%)	0.035	0.05–0.29	0.002
Horse	33	18 (54.5%)	Ref		
Sex					
Male	26	3 (11.5%)	Ref		
Female	32	16 (50.0%)	7.67	1.91–30.73	0.004
Age group					
≤3	14	6 (42.9%)	1.44	0.40–5.11	0.575
4‐10	35	12 (34.3%)	Ref		
>10	9	1 (11.1%)	0.24	0.03–2.14	0.202
Location					
Midlands	17	9 (52.9%)	3.97	1.05–14.89	0.041
Highlands	41	10 (24.4%)	Ref		
Respiratory disease					
Yes	33	16 (48.5%)	6.90	1.72–27.60	0.006
No	25	3 (12.0%)	Ref		
Total	58	19 (32.8%)			

OR: odds ratio; Ref: reference.

**TABLE 3:** Multivariable logistic regression analysis of risk factors associated with EHV‐5 detection

Variables	No. of equids	EHV‐5 positive (%)	OR	95% Confidence interval	*p* Value
Species					
Donkey	25	14 (56.0%)	5.73	1.75–18.75	0.004
Horse	33	6 (18.2%)	Ref		
Sex					
Male	26	10 (38.4%)	Ref		
Female	32	10 (31.3%)	0.73	0.25–2.16	0.566
Age group					
≤3	14	4 (28.6%)	0.87	0.22–3.41	0.845
4‐10	35	11 (31.4)	Ref		
>10	9	5 (55.6%)	2.73	0.61–12.17	0.189
Location					
Midlands	17	4 (23.5%)	0.57	0.14–2.39	0.444
Highlands	41	16 (39.0%)	Ref		
Respiratory disease					
Yes	33	11 (33.3%)	0.88	0.29–2.65	0.832
No	25	9 (36.0%)	Ref		
Total	58	20 (34.5%)			

OR: odds ratio; Ref: reference.

The proportion of positive equids for EHV‐2 and EHV‐5 was varied between male and female. EHV‐2 was detected in a significantly higher (*p* = 0.004) proportion of female (50%; *n* = 16) compared to male equids (11.5%; *n* = 3) (Table 2). In contrast, although not statistically significantly (*p* = 0.566), EHV‐5 was detected in a higher proportion of male (38.4%; *n* = 10) than female equids (31.3%; *n* = 10) (Table 3).

The detection of EHV‐2 and EHV‐5 varied for different age groups. EHV‐2 was detected in a higher proportion of yearlings and young equids (42.9%; *n* = 6) compared with adults (34.3%; *n* = 12) and old ages (11.1%; *n* = 1), however, the observed difference was not statistically significantly (*p* > 0.05) (Table 2). In contrast, although not significant (*p* > 0.05), EHV‐5 was detected in a higher proportion of older equids (55.6%; *n* = 5) compared with adults (31.4%; *n* = 11) and young equids (28.6%; *n* = 4) as shown in Table 3.

The proportion of EHV‐2 and EHV‐5 was compared between equids displaying clinical signs of respiratory disease (*n* = 33) and equids without clinical signs of illness (*n* = 25). EHV‐2 was detected in a significantly higher (*p* = 0.006) proportion of equids with signs of respiratory disease (48.5%; *n* = 16) compared to those without the disease (12%; *n* = 3). The odds of being positive for EHV‐2 in equids displaying clinical signs of respiratory disease was nearly 7 times higher than equids without showing clinical signs (Odds ratio (OR) = 6.9; 95% CI: 1.72–27.60) (Table 2). In contrast, EHV‐5 was detected in a slightly, but not significantly (*p* = 0.832), higher proportion in apparently healthy (36%; *n* = 9) equids compared to those displaying clinical signs of respiratory disease (33.3%; *n* = 11) (Table 3). Concurrent infection with EHV‐2 and EHV‐5 was found in 6 (10.3%) equids who exhibited respiratory clinical signs.

The proportion of equids infected with EHV‐2 and EHV‐5 varied within the different altitudes of the sampling sites. EHV‐2 was detected in a significantly higher (*p* = 0.041) proportion of equids living in midland (52.9%; *n* = 9) compared with highland (24.4%; *n* = 10). The odds of being positive for EHV‐2 in equids residing in the midlands was nearly four times higher than equids in the highlands (OR = 3.97; 95% CI: 1.05–14.89) (Table 2). In contrast, EHV‐5 was detected in a higher proportion of equids residing in highlands (39%; *n* = 16) compared with equids living in midland (23.5%; *n* = 4), however, this difference was not statistically significant (*p* = 0.444) (Table 3).

## 4 DISCUSSION

Equine respiratory infection is the most common problem that influences the performance of the working ability of equids. In Ethiopia, respiratory disease (coughing and nasal discharge) is the most common presenting complaint at veterinary clinics and a priority concern for owners of the working equids and veterinary practitioners. Equine herpesviruses are important pathogens that are involved in respiratory disease of varying severity. In the present study, detection of EHV‐2 and EHV‐5 from horses and donkeys in central Ethiopia is described. We also assessed the risk factors associated with respiratory disease and its clinical outcomes.

In the present study, from a total of 58 equids, 34.5% and 32.8% were found positive for EHV‐5 and EHV‐2, respectively. This shows that both EHV‐2 and EHV‐5 are prevalent in horses and donkeys residing in central Ethiopia. In this study, EHV‐5 was recorded relatively in a higher proportion than EHV‐2. This finding is consistent with previous studies conducted by Diallo et al. (2008) and Wang et al. (2007) in Australia and Negussie et al. (2017) in Ethiopia but in contrast to Dunowska et al. (2002) in New Zealand, Nordengrahn et al. (2002) in Sweden, Hungary and the United Kingdom and Torfason et al. (2008) in Iceland in which EHV‐2 has been recorded in a higher prevalence than EHV‐5.

In this study, EHV‐2 was detected in a significantly higher proportion of horses (54.5%) than donkeys (4%). In contrast, EHV‐5 was detected in a significantly higher proportion of donkeys (56%) as compared with horses (18.2%). With this study, it is difficult to establish a link between the detection of EHV‐2 and EHV‐5 with the host susceptibility. The existence of breed‐specific susceptibility differences (Stasiak et al., 2018) and species‐specific susceptibility differences (Negussie et al., 2017) has been reported. To validate host susceptibility variation, a more detailed investigation is needed using large sample sizes.

Co‐infection of EHV‐2 and EHV‐5 was found in 6 (10.3%) equids displaying clinical signs of respiratory disease. This dual infection is consistent with other reports (Nordengrahn et al., 2002; Allen and Murray, 2004; Ataseven et al., 2010; Negussie et al., 2017) in which both viruses can simultaneously infect equids. Co‐infection may have a synergistic effect on disease outcome by modulating host immune response and predisposing to secondary infections, which subsequently may lead to severe respiratory disease; however, it deserves further investigation.

EHV‐2 and EHV‐5 were found positive in 16 (48.5%) and 11 (33.3%) equids that exhibited clinical signs of respiratory disease, respectively. Similarly, EHV‐2 and EHV‐5 were tested positive from 3 (12%) and 9 (36%) equids with no signs of illness, respectively. The existence of EHV‐2 and EHV‐5 in apparently healthy equids is in agreement with previous studies conducted elsewhere (Ataseven et al., 2010; Laabassi al., 2017; Negussie et al., 2017; Stasiak et al., 2018). A significantly higher proportion of EHV‐2 was detected in equids with signs of respiratory disease (48.5%) as compared to those without clinical signs (12%). EHV‐2 positive equids were seven times more likely to display clinical signs of respiratory disease than EHV‐2‐negative equids (OR = 6.9; 95% CI: 1.72–27.60). The relationship between detection of EHV‐2 and displaying of clinical signs of respiratory disease may suggest the involvement of EHV‐2 in the development of respiratory diseases. Negussie et al. (2017) proposed that EHV‐2 may have a possible etiological contribution either to induce or predispose equids to respiratory diseases. Ataseven et al. (2010) stated that EHV‐2 played a pre‐disposing role in the occurrence of respiratory diseases. However, further detailed investigations are necessary to unequivocally conclude a causal association between EHV‐2 and the induction of clinical symptoms.

In this study, EHV‐2 was detected in a higher proportion in yearlings and young equids (42.9%; *n* = 6) as compared with adults (34.3%; *n* = 12) and old ages (11.1%; *n* = 1), however, the observed difference was not statistically significantly (*p* > 0.05). Our study is in agreement with previous studies where equids are highly infected with EHV‐2 in the first years of life despite the presence of maternal antibodies in the colostrums (Bell et al., 2006; Nordengrahn et al., 2002; Rushton et al., 2013). Similarly, although not significant (*p* > 0.05), EHV‐5 was detected in a higher proportion in older equids (55.6%; *n* = 5) as compared with adults (31.4%; *n* = 11) and young (28.6%; *n* = 4) (Table 3). Some epidemiologic evidence suggests that foals are usually infected with EHV‐5 later on in life than with EHV‐2 (Bell et al., 2006; Nordengrahn et al., 2002).

In this study, differences in the proportion of equids positive for EHV‐2 and EHV‐5 were observed between males and females. EHV‐2 was detected in a significantly higher proportion of females compared to male equids. This is in agreement with the previous report by Negussie et al. (2017), who reported a higher prevalence of EHV‐1, EHV‐2 and EHV‐5 in females than in males. The possible reason might be due to stress‐induced immunosuppression in mares during foaling, weaning, pregnancy and lactation, which subsequently results in reactivation of latent equine herpesviruses and viral shedding, but this requires further investigation.

The proportion of equids infected with EHV‐2 and EHV‐5 was varied in different geographical settings. A significantly higher (*p* = 0.041) proportion of EHV‐2‐test‐positive equids was found in midlands (52.9%) as compared with equids residing in highlands (24.4%). The odds of being positive for EHV‐2 in equids residing in the midlands was nearly four times higher than equids in the highlands (OR = 3.97; 95% CI: 1.05–14.89). This study suggests a wide distribution and varying susceptibility to the viruses in different agro‐ecology. Currently, there is little evidence to support an association between altitudes with gammaherpesvirus infection. Azab et al. (2019) reported equid herpesviruses prevalence variation attributed to geographical variability and environmental factors. However, at present, a possible explanation of why equids residing in different altitudes have varying susceptibility to gammaherpesvirus infection could not be given, thus more work is needed to elucidate the exact relationship.

In conclusion, EHV‐2 and EHV‐5 were detected both in horses and donkeys residing in central Ethiopia. Concurrent infection with EHV‐2 and EHV‐5 was found in those equids that exhibited respiratory clinical signs. The association between EHV‐2‐test‐positive equids and displaying of clinical signs of respiratory disease was observed, which suggests EHV‐2 involvement in the development of respiratory disease; however, it deserves further investigation.

## AUTHOR CONTRIBUTIONS

KW performed field sample collection, laboratory analysis, and wrote the draft manuscript. SL participated in data analysis. KA analysed the data and participated in reviewing the manuscript. HN set up the study designs, participated in sample collection and laboratory analysis, coordinated the work, and wrote the manuscript. All authors read and approved the final manuscript.

## COMPETING INTERESTS

The authors declare that they have no competing interests.

## FUNDING

This study was supported by the Addis Ababa University thematic research fund (VPRTT/PY‐096/2018), Ethiopia. The funder had no role in the conception, design of the study, data collection, analysis and interpretation of the data reported in this manuscript.

## ETHICS DECLARATIONS

Ethical approval for this study was granted from the animal research ethical review committee of the College of Veterinary Medicine and Agriculture of the Addis Ababa University (Reference number: VM/ERC/08/01/12/020). All methods were performed in accordance with relevant guidelines and regulations. All protocols were approved by the animal research ethical review committee. Before conducting the research, equine owners were informed with the objectives and the benefits of the study and they gave consent for their animal's inclusion in the study. The informed consent was obtained from all participating equine owners before sample collection and this was approved by the ethics committee. The consent was verbal because they are unable to write and read. These consents were taken in the presence of a third independent party.

## CONSENT FOR PUBLICATION

Consent to publish the finding of the data was obtained verbally from all equine owners during sampling and data collection.

## Data Availability

The datasets used during the current study are available on request from the corresponding author.
